# Study on policy adaptability of occupational injury insurance for employees of emerging business sectors in China

**DOI:** 10.3389/fpubh.2025.1646962

**Published:** 2025-09-02

**Authors:** Qixia Du, Xinru Miao, Xiaoman Cui, AiJun Xu, Xuebin Qiao

**Affiliations:** ^1^School of Health Economics and Management, Nanjing University of Chinese Medicine, Nanjing, China; ^2^School of Elderly Care Service and Management, Nanjing University of Chinese Medicine, Nanjing, China; ^3^School of Social and Public Administration, East China University of Science and Technology, Shanghai, China; ^4^School of Business, Nanjing Audit University, Nanjing, China

**Keywords:** emerging business sectors, occupational injury insurance, policy instruments, policy intensity, policy stakeholders, policy adaptability, policy optimization

## Abstract

**Introduction:**

With the rapid industrialization in China, the issue of labor rights protection in emerging business sectors has become a hot topic within academic research and market practice. However, there were still few studies on the assessment of policy instruments in China. Accordingly, this study attempted to construct a three-dimensional framework regarding “policy instruments, policy intensity, and policy stakeholders,” to explore the policy adaptability of occupational injury insurance for employees of emerging business sectors in China.

**Methods:**

Followed by compiling 60 policies pertaining to occupational injury insurance for employees of emerging business sectors in China, this study conducted Nvivo 12 to summarize the specific provisions of these policies. Subsequently, we employed the rule code of “policy serial number, chapter serial number, and clause serial number” to investigate the adaptability of these occupational injury insurance policies.

**Results:**

It suggested that the development of occupational injury insurance policies for employees of emerging business sectors in China was still in its early stages, with characteristics of uneven utilization of policy instruments and limited adaptability to specific targets. While there was an upward trend in policy intensity, improvements in efficiency and capacity were necessary. Moreover, the presence of competing policy interests and inadequate governance capacity remained significant challenges.

**Discussion:**

Based on the central theme of “content adaptation, effective instruments, and inclusive development,” this study attempted to provide several managerial implications. First, it should enhance the publicity of policies to increase policy satisfaction. Second, it was necessary to refine planning to improve the efficiency of public policies. Third, there was a need to optimize the application structure of policy tools to enhance the alignment with policy goals. Finally, it needed to give priority to the needs of emerging business sectors to ensure a balance between policy supply and demand.

## 1 Introduction

With the rapid technological progress and industrial innovation in China, a growing number of emerging business sectors have emerged in China (1). Data from the 9th National Survey on the Workforce Status demonstrated that, as of March 2023, emerging business sectors have become a significant component of the workforce, encompassing approximately 84 million employees. In May 2024, the Ministry of Human Resources and Social Security announced that China officially identified 19 new professions, including application personnel of generative artificial intelligence system, testers of intelligent connected vehicles, operation and maintenance personnel of intelligent manufacturing system, and operation and maintenance personnel of industrial Internet. This indicates that emerging business sectors are making critical contributions to job creation and social progress in the contemporary China.

Along with the turbulent changes in emerging business sectors and the rapid expansion of the labor market, intensified competition among employment personnel is likely to lead to increased work pressure and potential risk factors, such as blurred boundaries between work and leisure time, inadequate occupational health training, unstable income, and insufficient social welfare provisions (2). In this regard, there is an urgent need to develop labor security frameworks. Moreover, it is not only a powerful guarantee for the individual rights of employees of emerging business sectors, but also an important support for the development of new quality productivity.

In terms of policy practice, China is concentrating on exploring the establishment and implementation of an occupational injury insurance system for employment personnel. For instance, as early as 2016, the “Thirteenth Five Year Plan Outline for the Development of Human Resources and Social Security” proposed the need to actively explore work injury insurance methods suitable for employment personnel. At the beginning of 2020, the No. 1 central document of the Chinese Central Government also explicitly mentioned the need to carry out the pilot project of occupational injury protection for employees in emerging business sectors. In December 2021, the Ministry of Human Resources and Social Security of China and 10 other ministries jointly issued a notice on the pilot work of occupational injury protection for employment personnel in emerging business sectors, proposing to select some large-scale travel, food delivery, instant delivery, and local freight platform enterprises to carry out pilot work on occupational injury protection for employment personnel. Since July 1, 2022, the Ministry of Human Resources and Social Security of China, together with relevant ministries, has launched a pilot program for occupational injury protection for employment personnel in some provinces and platform enterprises, and thus the coverage of the system has steadily improved. According to the Ministry of Human Resources and Social Security of China, as of September 2023, a total of 6.68 million individuals have been encompassed under occupational injury protection, and the pilot objectives have largely achieved comprehensive coverage.

Accompanied by the rapid expansion of employee number within emerging business sectors, the deficiencies in occupational injury insurance for employees of emerging business sectors in China have increasingly drawn the attention of scholars. In the Chinese literature, some studies argued that a novel operational framework for occupational injury insurance should be established to develop a fully socialized insurance model. Bai (3) proposed the development of a multidimensional occupational injury protection system for employees of emerging business sectors, facilitated by multi-agent participation and interaction. Based on the analysis framework of the Hand formula, Yu (4) posits that the prevention costs incurred by the platform are generally lower than those borne by employees. While occupational injuries are attributed to the platform, the costs associated with employees fulfilling their necessary prevention obligations are lower than the potential damages incurred. Yue (5) contended that the pilot areas exhibit varying levels of development and significantly different system designs, which pose substantial obstacles to the future implementation of a unified new form of employment at the national level.

However, insufficient attention has been given to the adaptability of pilot policies and objectives across different levels of government, and no unified countermeasures at the national level have been explored from the perspective of collaborative governance. In this regard, it needs to investigate the adaptability of occupational injury insurance policies for employees in emerging business sectors. Accordingly, this study attempts to construct a framework of mandatory, voluntary, and hybrid policy instruments based on policy texts analysis of occupational injury insurance to explore the adaptability of these instruments and their objectives, and then propose some pathways for further optimization.

## 2 Theoretical analysis

### 2.1 The theory of policy adaptability

In general, the theory of policy adaptability is often used to describe the matching degree between policies, specific environment, target groups, and implementation conditions (6, 7). According to previous literature, this study analyzes the degree of policy adaptability in occupational injury insurance for employees of emerging business sectors from three dimensions, including policy instruments, policy intensity, and policy stakeholders (8).

First of all, the adaptability of existing policies within the social and economic structure is evident through the examination of policy instruments (9), as well as an assessment of the capacity and resources of the social insurance system to implement occupational injury insurance. The extent of policy intensity can be analyzed to determine whether occupational injury insurance policies encounter macro-environmental support and constraints during the implementation process (10). From the perspective of stakeholders, analysis of the adaptability of policies should primarily consider whether the formulation of policies aligns with the actual needs of employees in emerging business sectors (11). Furthermore, it needs to examine whether stakeholders have been able to actively promote policy implementation throughout the process. The degree of alignment between policy utilization and actual insurance willingness, as well as supply and demand, can be assessed through policy instruments.

### 2.2 Policy adaptation criteria

It is widely acknowledged that the economic and social development levels in China vary across different geographic locations. Meanwhile, employees in the Chinese emerging business sectors exhibit diversified characteristics such as significant increase in number, strong mobility, and widespread distribution (12). According to the theory of policy adaptability, therefore, the introduction of occupational injury insurance policies for employees in emerging business sectors should address the dynamic and differentiated needs of various regions. Through continuous optimization of policies, it may contribute to achieving compatibility between policy supply and actual demand, ultimately fulfilling the intended policy objectives.

First, in terms of policy instruments, it can facilitate the adaptation of the “functional goals.” Whether adopting voluntary, hybrid, or mandatory policy instruments, these serve as means and approaches of government governance, acting as a bridge between public policy goals and outcomes (13). Therefore, their policy functions need to be aligned with strategic objectives.

Second, in terms of policy intensity, it is crucial to ensure the adaptability of policy effectiveness levels to capacity issues (14). With the advancement of new forms of employment, the effective supply of the occupational injury insurance system is insufficient, and the shortage of occupational injury insurance rights for employees in these new forms of employment is particularly notable. If policies are not set in a timely manner or are handled improperly, they can bring about deeper contradictions. Therefore, it is necessary to build a policy capability system that aligns with the current problems.

Third, in terms of policy stakeholders, it should address the adaptation of “synergy alienation” (15). In judicial practice, the judgment results often lead to complex disputes among employees, platform enterprises, affiliated enterprises, and other related groups, complicating the establishment and coordination of multiple responsible parties’ participation in social insurance. Consequently, it is necessary to continuously improve policy design, coordinate the behaviors of policy stakeholders, and achieve the coordinated adaptability of policies and behaviors. Furthermore, it is imperative to address the shortcomings in response to the practical difficulties in implementing occupational injury insurance policies for employees of emerging business sectors.

## 3 Construction of the research framework

The policy of occupational injury insurance for employees in emerging business sectors can be characteristics with clear targets and objectives, but its implementation mechanism is still unclear. Therefore, we need to examine several key issues: (1) whether occupational injury insurance includes work-related injury insurance or commercial insurance; (2) how to accurately define employee identity of emerging business sectors; and (3) how to clarify the attribution of occupational injury liability. From the perspective of the necessity for high-quality development of social security and the reality of strengthening the advancement of emerging business sectors, there are some challenges in the construction of occupational injury insurance for employees in emerging business sectors in China. Its essential characteristics are evident in the “non-standardization of labor relations,” “non-regularization of labor employment,” and “diversification of labor injury risks.” In this regard, it is crucial to consider the compatibility among various supporting factors, including financial support, public services, risk prevention, regulatory oversight, and the survival needs of employees.

**Figure 1:**
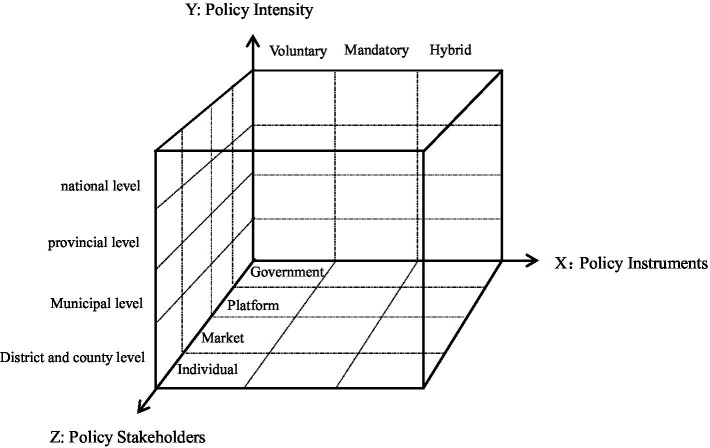
A three-dimensional analytical framework for occupational injury insurance policies applicable to employees in emerging business sectors.

Based on a comprehensive understanding of the construction mechanism of occupational injury insurance for employees of emerging business sectors, this study aims to develop an adaptive research framework. Derived from an analysis of policy text content, this study posits that policy goal-instrument adaptability refers to the degree of alignment between the connotation of policy instruments and actual development needs (16). The policy goal-instrument analysis should address the issue of policy instrument utilization and consider the various policy forces and stakeholders involved (17). As shown in Figure 1, consequently, this study presents a three-dimensional theoretical analysis framework comprising policy instruments, policy intensity, and policy stakeholders, to explore the current development status, existing challenges, and potential optimization pathways for the adaptability of occupational injury insurance policies for employees of emerging business sectors in China. In this framework, the X dimension is defined as the type of policy instruments, the Y dimension as policy intensity, and the Z dimension as stakeholders. This framework will be utilized to analyze both the general situation regarding the use of policy instruments in occupational injury insurance for employees of emerging business sectors and the strategies for selecting policy instruments under varying policy intensity and stakeholder dynamics.

### 3.1 X dimension: policy instruments

In accordance with the theory of policy adaptability, the selection of policy instruments is directly related to the achievement of policy goals. Since the 1990s, the study on policy instruments has gradually become a hot topic in policy research. Previous literature argued that policy instruments can be categorized into various types. As the most representative classification, voluntary, mandatory, and hybrid policy instruments reflect the different degrees of government intervention in the processes of public goods and services (18).

Characterized by being rarely influenced by the government, voluntary instruments include families, communities, voluntary organizations, and the market (19). As known as direct tools, mandatory instruments act on target individuals or organizations in a compulsory or direct manner, including regulation, public enterprises, and direct provision. Mixed instruments combine features of both voluntary and mandatory instruments, including information and persuasion, subsidies, property rights auctions, taxation, and user fees. The definitions of theses policy instruments are outlined in Table 1.

**Table 1:** Policy types, instrument names, and their descriptions.

Policy types	Instrument names	Descriptions
Voluntary policy instruments	Family and community	The assumption of responsibility for individual occupational injuries by families and communities on a voluntary basis.
	Volunteer Organization	Public services for occupational injury protection are provided by non-profit civil society organizations.
	Market	Individuals are free to choose occupational injury insurance in the market and enjoy the corresponding protection.
Mandatory policy instruments	Regulation	The government requires individuals or organizations to participate in occupational injury insurance.
	Public enterprise	The government provides public services through state-owned enterprises.
	Direct delivery	The government provides occupational injury insurance directly to new industries with the support of public finance.
Hybrid policy instruments	Information and exhortation	The government communicates to the relevant authorities its willingness to provide occupational injury protection for people working in new sectors.
	Subsidy	The government provides various forms of occupational injury subsidies to individuals, companies and other NGOs.
	Property auction	The government establishes a fixed number of transferable property rights and creates a market for resources that are not scarce.
	Taxes and user fees	Statutory individuals or platforms pay taxes at various rates in accordance with the law, and the insured platforms pay occupational injury insurance premiums to the government.

### 3.2 Y dimension: policy intensity

Policy intensity reflects the government’s emphasis on the significance of a given policy, while the magnitude of this intensity serves as a key factor in determining the effectiveness of regulation (20, 21). Based on the levels of administrative power institutions in China, the rankings of policy intensity from highest to lowest are as follows: the issuance of documents by the State Council ministries and commissions, provincial-level local regulations, municipal-level local regulations, and district (county)-level local regulation. As shown in Table 2, the higher the score is, the greater the policy intensity is.

**Table 2:** Quantitative standards for policy intensity in occupational injury insurance for employees in emerging business sectors.

Policy intensity score	Quantitative standards
4	State Council ministries and commissions issued documents
3	Provincial local regulations
2	Municipal local regulations
1	District or county local regulations

### 3.3 Z dimension: policy stakeholders

The nature of public policy involves the strategies employed by decision-making entities, particularly the government, to utilize their granted public power to identify social interest needs and coordinate social conflicts (22). Specifically, public policy serves as a crucial means of coordinating the relationships among the government, market, society, and individuals. The selection of policy instruments reflects the extent of transformation in policy functions and government service reform during the paradigm shift in the governance of public problems. The occupational injury insurance policy for employees in emerging business sectors entails multiple stakeholders, including the government, employers, employment platforms, and individuals. The implementation approaches of pilot insurance policies elucidate the government’s concepts and methods of governance regarding the occupational injury risks faced by employees of emerging business sectors.

## 4 Research methodology

### 4.1 Data sources

To ensure representativeness of data, this study sought information on occupational injury protection for employees in emerging business sectors from official websites of the State Council, provinces and cities. After conducting searches using keywords such as “emerging business sectors” and “occupational injury insurance,” a total of 60 representative policy documents have been selected (as shown in Table 3).

**Table 3:** Summary of representative policy documents.

Number	Policy documents	Release time	Document number
1	Notice on the Participation of Flexibly Employed Persons in Work-Related Injury Insurance	2006	Tonglao Social Worker [2006] No. 14
……	……	……	……
60	Measures for Participation in Work-Related Injury Insurance for Specific Persons Recruited by Employers in Zhejiang Province Who Are Not in Compliance with the Situation of Establishing a Labour Relationship (for Trial Implementation)	2023	Zhejiang Province People’s Social Development [2023] No. 21

### 4.2 Policy coding framework

Using NVivo 12, this study summarizes the specific contents of occupational injury insurance for employees of emerging business sectors, which were listed in 60 policy documents. By employing the “policy serial number, chapter serial number, and clause serial number” as coding rules, this study generates a sample code reflecting the content of occupational injury insurance for employees of emerging business sectors (Table 4).

**Table 4:** Examples of sample coding for occupational injury insurance content for employees in emerging business sectors.

Number	Policy documents	Element	Coding	Policy instruments
1	Notice on the Participation of Flexibly Employed Persons in Work-Related Injury Insurance	The amount of the fee is determined in accordance with the principle of “setting revenue on the basis of expenditure and balancing income and expenditure,” and is included in the management of the Industrial Injury Insurance Fund.	1-2-1	Hybrid policy instruments
……	……	……	……	……
60	Measures for Participation in Work-Related Injury Insurance for Specific Persons Recruited by Employers in Zhejiang Province Who Are Not in Compliance with the Situation of Establishing a Labor Relationship (for Trial Implementation)	Provincial departments of human resources and social security, finance and taxation may adjust the applicable scope of objects and contribution standards in due course according to the trial implementation.	60-1-8	Mandatory policy instruments

### 4.3 Analysis of policy texts

By utilizing Excel to analyze the coding content, as shown in Table 5, we found that there are a total of 281 types of mandatory policy instruments, accounting for 73.75%. Meanwhile, there are 36 types of voluntary policy instruments (9.45%) and 64 types of hybrid policy instruments (16.80%).

**Table 5:** Statistical analysis of policy instrument nodes.

Policy types	Instrument names	Node number	Proportion	Total
Voluntary policy instruments	Family and community	2	5.56%	9.45%
	Volunteer Organization	13	36.11%	
	Market	21	58.33%	
Mandatory policy instruments	Regulation	271	96.44%	73.75%
	Public enterprise	0	0.00%	
	Direct delivery	10	3.56%	
Hybrid policy instruments	Information and exhortation	9	14.06%	16.80%
	Subsidies	18	28.13%	
	Property auction	7	10.94%	
	Taxes and user fees	30	46.88%	

**Figure 2:**
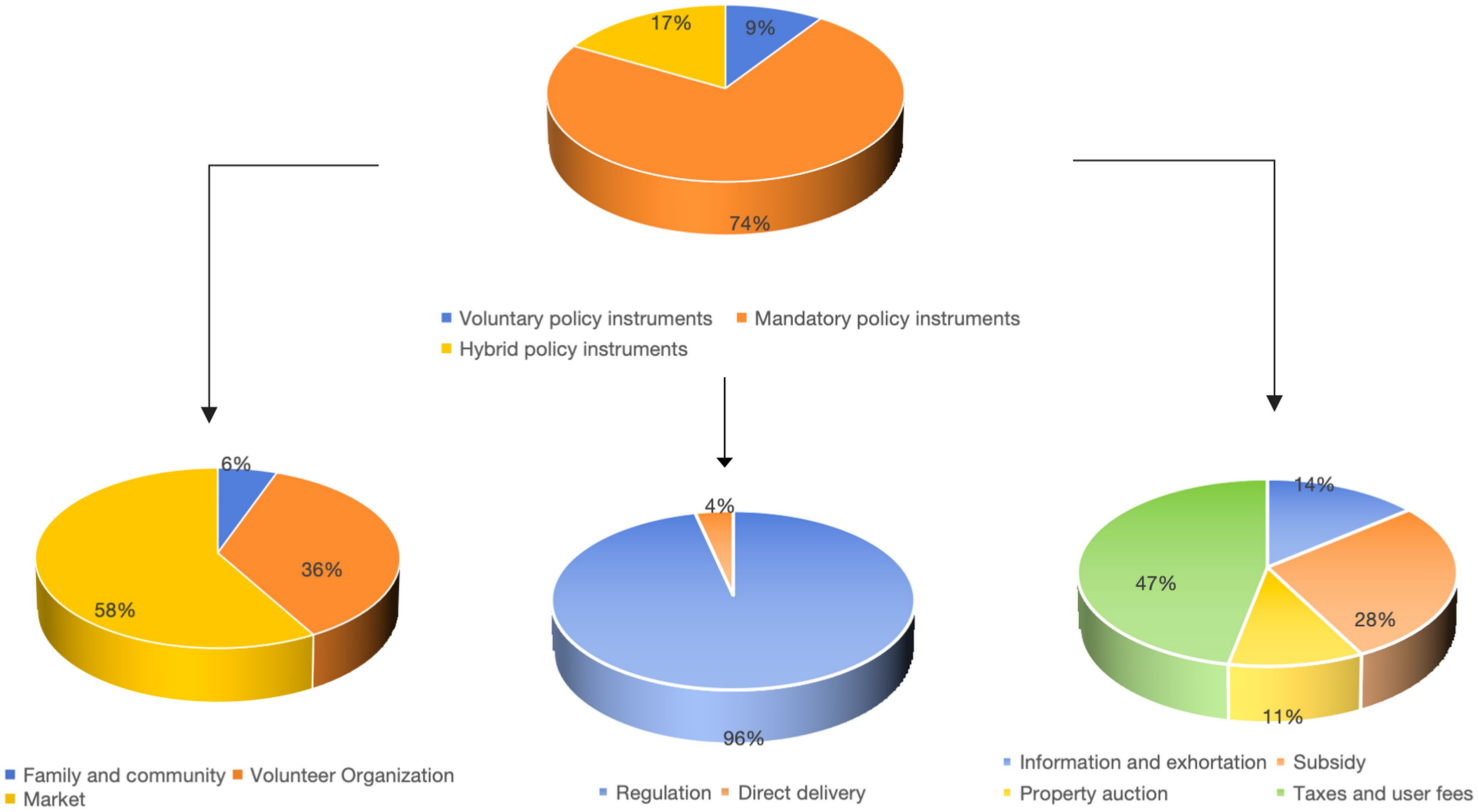
Proportion of the usage of policy instruments.

As shown in Figure 2, mandatory policy instruments represent the highest proportion, followed by hybrid policy instruments, with voluntary policy instruments representing the lowest proportion, indicating a significant overall imbalance. Among the various types of voluntary policy instruments, the market accounts for the highest proportion at 58.33%, comprising a total of 21 corresponding policy codes. The second highest proportion is attributed to voluntary organizations, which account for 36.11%, while families and society represent the lowest proportion at 5.56%. Among the categories of mandatory policy instruments, regulatory policy instruments constitute the highest proportion at 96.44%, with a direct provision of 3.56%. Additionally, policy instruments of public enterprise are not utilized. Among the hybrid policy instrument types, user fees and taxes account for the highest proportion at 46.88%, while information and advice account for 14.06%, property auctions account for 10.94%, and subsidies account for 28.13%.

#### 4.3.1 The utilization of voluntary policy instruments

Voluntary policy instruments are tools provided by the market, families, communities, and volunteer organizations on a voluntary basis to mitigate or diversify the occupational injury risks faced by employees in emerging business sectors, encompassing a total of 36 codes. While constructing an occupational injury protection system for employees in emerging business sectors, it should fully emphasize the concept of multi-party joint governance, enabling the market, families, society, and volunteer organizations to actively fulfill their obligations to protect the rights of employees (23). Simultaneously, it is important to fully respect the wishes of workers and encourage employees of emerging business sectors to actively participate in occupational injury insurance. However, in comparison to other countries, the use of voluntary policy instruments in China is relatively limited (24), indicating that the framework for occupational injury insurance is still inadequate. Therefore, it is necessary to continuously update and enhance policy formulation to better align with current realities.

Specifically, the market has the largest number of codes among voluntary policy instruments. In policy documents, the government has consistently put suggestions for employment platforms, encouraging to diversify the occupational injury risk burden of employees of emerging business sectors through the purchase of commercial insurance. Therefore, the market serves as the primary vehicle for voluntary policy instruments. Volunteer organizations primarily focus on the government’s suggestions directed at trade unions and social organizations, promoting the recruitment of individuals in new forms of employment to join these organizations, establish funds, and provide assistance. Through the collective power of these groups, they play a protective role in supporting new forms of employment and their employees. Families and society primarily rely on grassroots organizations, utilizing the registration information of community organizations to facilitate participation in occupational injury insurance and to enhance outpatient and community medical rehabilitation efforts.

#### 4.3.2 The utilization of mandatory policy instruments

Mandatory policy instruments represent the highest level of government intervention in occupational injury insurance for employees of emerging business sectors through direct provision, legal regulations, or other mechanisms (25). As shown in Table 5, the largest number of policy codes utilized for regulatory policy instruments is 271, which includes mandates for employers to provide work-related injury insurance for their part-time employees. Outsourcing companies are required to employ workers in accordance with applicable laws and regulations, and those engaging in illegal employment must face legal responsibilities. By adopting a combination of government leadership, information technology guidance, and social responsibility, we aim to construct operational mechanisms for occupational injury protection management services. The protection of the rights of employees in emerging business sectors is still in a state of continuous development. Therefore, the government needs to mandate relevant departments, employment platforms or enterprises, and individuals to gradually establish occupational injury insurance systems for employees of emerging business sectors through mandatory legal and policy provisions (26, 27). In the case of delivering policy instruments, the government primarily employs a pilot model to implement occupational injury insurance institutions for new types of employees in designated pilot areas, following the process of “pilot, promotion, and scaling” to foster the development of the platform economy while reasonably protecting the rights of employees.

Obviously, it suggests that government departments attach significant importance to the protection of the rights of employees in new forms of employment, with a particular emphasis on occupational injury protection for employees (28). By employing mandatory policy instruments that require platforms to actively participate in employee insurance and require government agencies to standardize the processes for work-related injury recognition, they strive to establish norms across various links and aspects. This approach aims to optimize social security services for new forms of employment (29).

#### 4.3.3 The utilization of hybrid policy instruments

Hybrid policy instruments constitute policy guidance initiated by the government while fully considering the interests of both individuals and enterprises (30, 31). Among the three types of policy instruments, this category represents the smallest proportion, accounting for only 16.8%. In contrast, the degree of alignment between policies and reality during the actual implementation process is often underestimated, and the experiences of individuals and enterprises during the implementation of new occupational injury insurance formats are frequently overlooked.

Generally speaking, hybrid policy instruments encompass information and advice, subsidies, property auctions, taxation, and user fees (32, 33). The highest proportion is taxes and user fees, primarily because the government has stipulated the fees for occupational injury insurance or work-related injury insurance to be paid by platforms. Subsidies account for the second-largest proportion, as the government provides compensation and subsidies to employees who have suffered occupational injuries. Property auctions involve platforms outsourcing insurance policies to employees in emerging business sectors. The human resources and social security bureaus of many cities determine commercial insurance institutions through open bidding, which not only disperses the pressure on platform employment and government protection but also provides profit opportunities for insurance institutions. Additionally, information and persuasion serve as tools for the government to encourage platforms to explore security models, thereby ensuring the freedom and diversity of enterprise insurance.

In sum, the government has directed attention toward the emerging business sectors and adopted a diversified approach to provide comprehensive protection in conjunction with the market. By connecting all parties and ensuring the interests of multiple stakeholders, the information gap has been narrowed, and the level of occupational injury insurance protection for employees in the new industry has been enhanced.

### 4.4 Analysis of policy stakeholders

**Figure 3:**
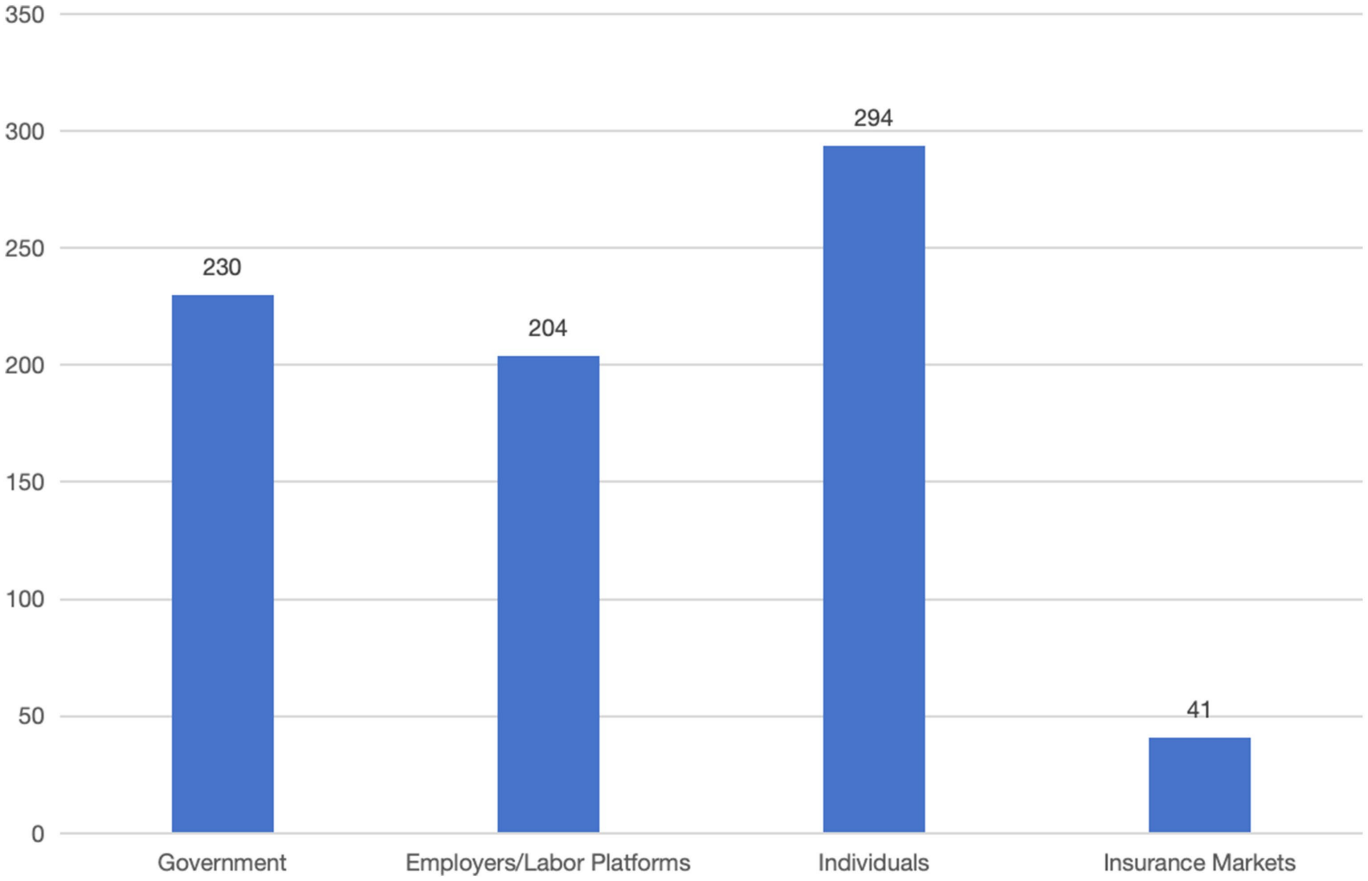
Statistical bar chart of stakeholders of occupational injury insurance for employees of emerging business sectors.

As shown in Figure 3, among the 381 policy codes, 294 codes are directly or indirectly related to personal interests, constituting 77.1%. There are 204 policy codes pertaining to employment platforms or employers, accounting for 53.5%, and there are 230 government policy codes, accounting for 60.3%. Additionally, there are 41 insurance market policy codes, constituting 10.7%.

The concentration of policy stakeholders on individuals reflects that national policy regarding occupational injury insurance for new types of employees has consistently revolved around the interests of insured individuals, thereby ensuring the effective implementation of policy tools aimed at these individuals. Moreover, employment platforms and enterprises directly assume the obligation of occupational injury protection for employees of emerging business sectors during the actual implementation process. Consequently, multiple policies have clearly defined regulations for enterprises and platforms, providing appropriate preferential treatment in payment rules. This framework encourages them to address the occupational risks that the government and the market must bear, thereby motivating enterprises and platforms to actively protect the legitimate rights and interests of employees.

Besides individuals and platform enterprises, regional governments are also important policy stakeholders. They respond to national policies, learn from the experiences of pilot cities, and improve the guarantee system. Consequently, they may enhance both the strength of guarantees and administrative efficiency. When new forms of employment result in occupational injuries and labor disputes, the government not only needs to provide financial assistance but also needs to actively coordinate with judicial and administrative departments to address these issues (34).

Additionally, the insurance market is also an important stakeholder that plays a significant role in the development of occupational injury insurance for new types of employees.

The market has achieved high levels of utilization and participation in the process of transitioning employment insurance, converting enterprise platforms to commercial insurance, and introducing market-oriented insurance mechanisms by the government. Therefore, the market is also required to bear increased risks regarding occupational injury.

### 4.5 Analysis of policy intensity

**Figure 4:**
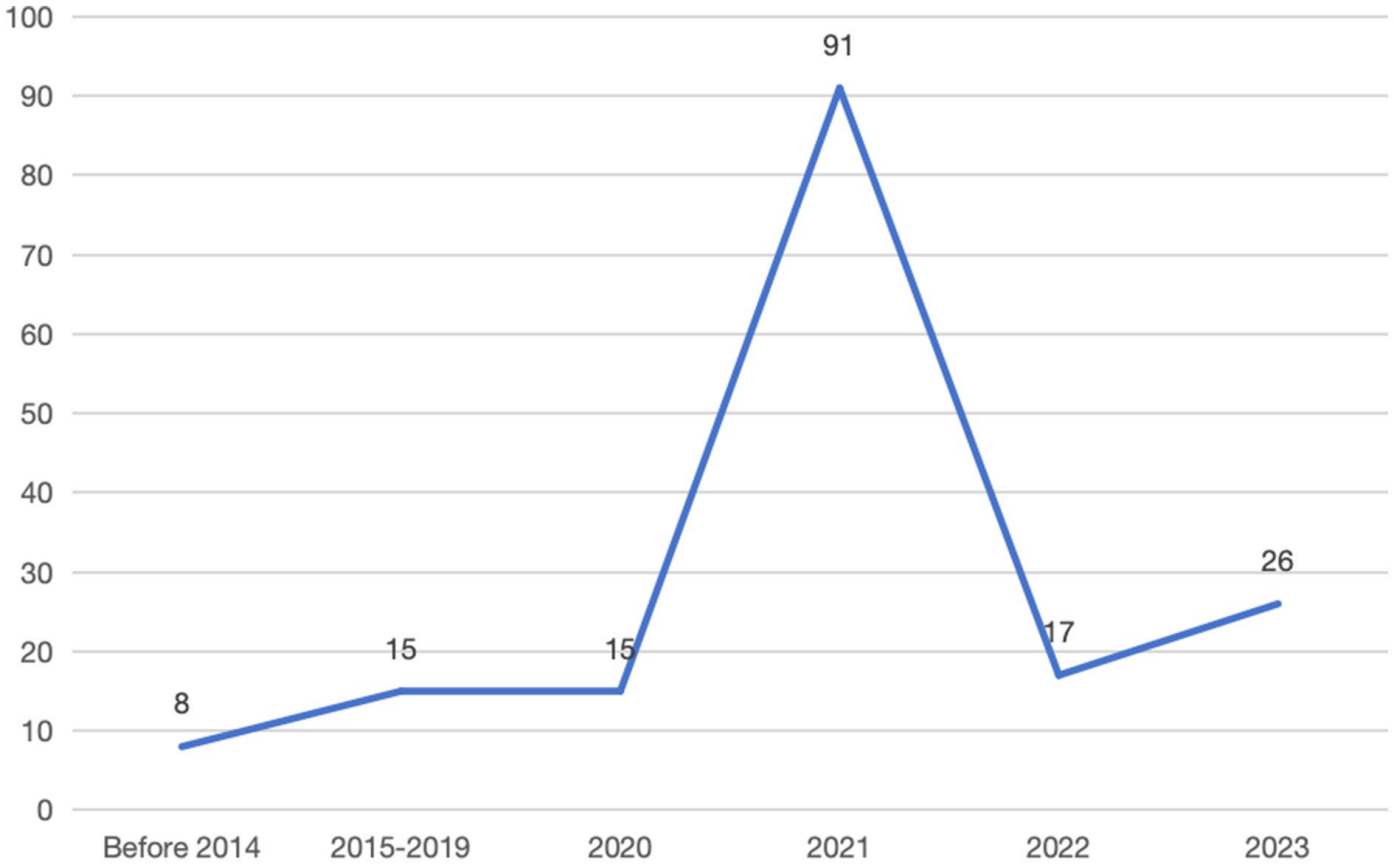
Line chart of total policy intensity levels.

The evolution of occupational injury insurance policies can be divided into three stages. Prior to 2014, policy issues had not yet materialized. From 2015 to 2019, policy issues began to emerge. Since 2020, occupational injury protection for flexible employment personnel on platforms has become a priority issue. Accordingly, this study attempts to examine these three stages and focus on refining the policy timeline after 2020. By analyzing the time periods and time points (Figure 4), the overall intensity of the policy documents is determined. Through comparisons of the intensity levels of the collected policy documents, we found that the policy intensities before 2014, from 2015 to 2019, 2020, 2021, 2022, and 2023 were 11, 12, 15, 91, 17, and 26, respectively. It implied that the overall policy intensity level has fluctuated, peaking in 2021.

With the rapid changes of new forms of employment, the number of individuals opt for these alternatives is growing, particularly in the express delivery and food delivery industries. As a result, employment-related issues are gradually being amplified, and the contradictions in occupational security caused by labor relations are also intensifying. Therefore, over the past 5 years, the government has placed greater emphasis on the newly employed population, gradually introducing relevant policies to address various challenges that arise in the workplace, constantly reinforcing the responsibilities of both enterprises and governments, and enhancing the protection of new forms of employment.

Before 2014, the Chinese central government did not provide adequate occupational injury protection for employees in emerging business sectors, and only sporadically addressed this issue within the framework of work-related injury insurance, while local governments proposed their regional regulations. From 2015 to 2019, the issue of occupational injury protection for employees in emerging business sectors began to gain attention, leading the central government to gradually focus on these employment groups and introduce several policies. In 2020, the State Council enacted relevant policies to direct the development of new economic forms, specifically addressing the protection of the rights of employees at the platform level. Following the issuance of opinions by the General Office of the State Council regarding new forms of employment, various government agencies and local authorities responded to the national call by enacting detailed regulations to support these employment models and safeguard the rights of employees.

In addition, increased social attention and the significant number of occupational injuries caused by new forms of employment have led to the peak of policy intensity in 2021. In 2022, there was a decline in policy intensity due to local departments continuing to implement the guidance and implementation opinions from 2021, but the overall development was positive. In 2023, the central government planed to focus on developing high-quality work injury rehabilitation services, and further strengthened the occupational injury insurance policies for new types of employees.

## 5 Findings

### 5.1 X-Y cross analysis

**Figure 5:**
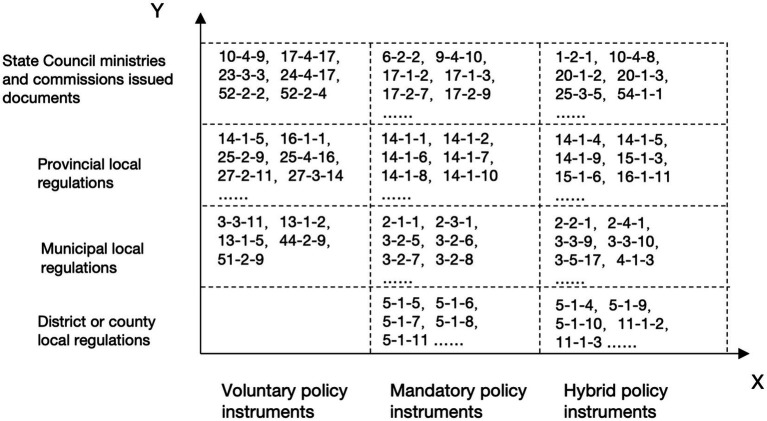
X-Y cross analysis encoding diagram.

Through the X-Y cross analysis, specifically the two-dimensional analysis of policy instrument intensity, it can elucidate the relationship between the use of policy instruments and their effectiveness (35). The relevant code was illustrated in Figure 5, and the specific values were demonstrated in Table 6. Through vertical and horizontal comparisons within the tables, we can conclude that the effectiveness of policy instruments is uneven, and their application across different levels of policy intensity also demonstrates significant variability. Mandatory policy instruments remain predominant, accounting for 66.67, 72.5, 75.12, and 72.92% of local regulations at the district or county level, city level, provincial level, and state level, respectively.

**Table 6:** Statistics of X-Y cross analysis.

Policy types	Instrument names	District or county local regulations	Municipal local regulations	Provincial local regulations	State Council ministries and commissions issued documents	Total
Voluntary policy instruments	Family and community	0	0	0	2	2
	Volunteer Organization	0	0	11	2	13
	Market	0	5	14	2	21
Mandatory policy instruments	Regulation	8	84	144	35	271
	Public enterprise	0	0	0	0	0
	Direct delivery	0	3	7	0	10
Hybrid policy instruments	Information and exhortation	0	2	4	3	9
	Subsidies	1	11	6	0	18
	Property auction	1	2	4	0	7
	Taxes and user fees	2	13	11	4	30
Total		12	120	201	48	381
Percentage		100%	100%	100%	100%	100%

**Figure 6:**
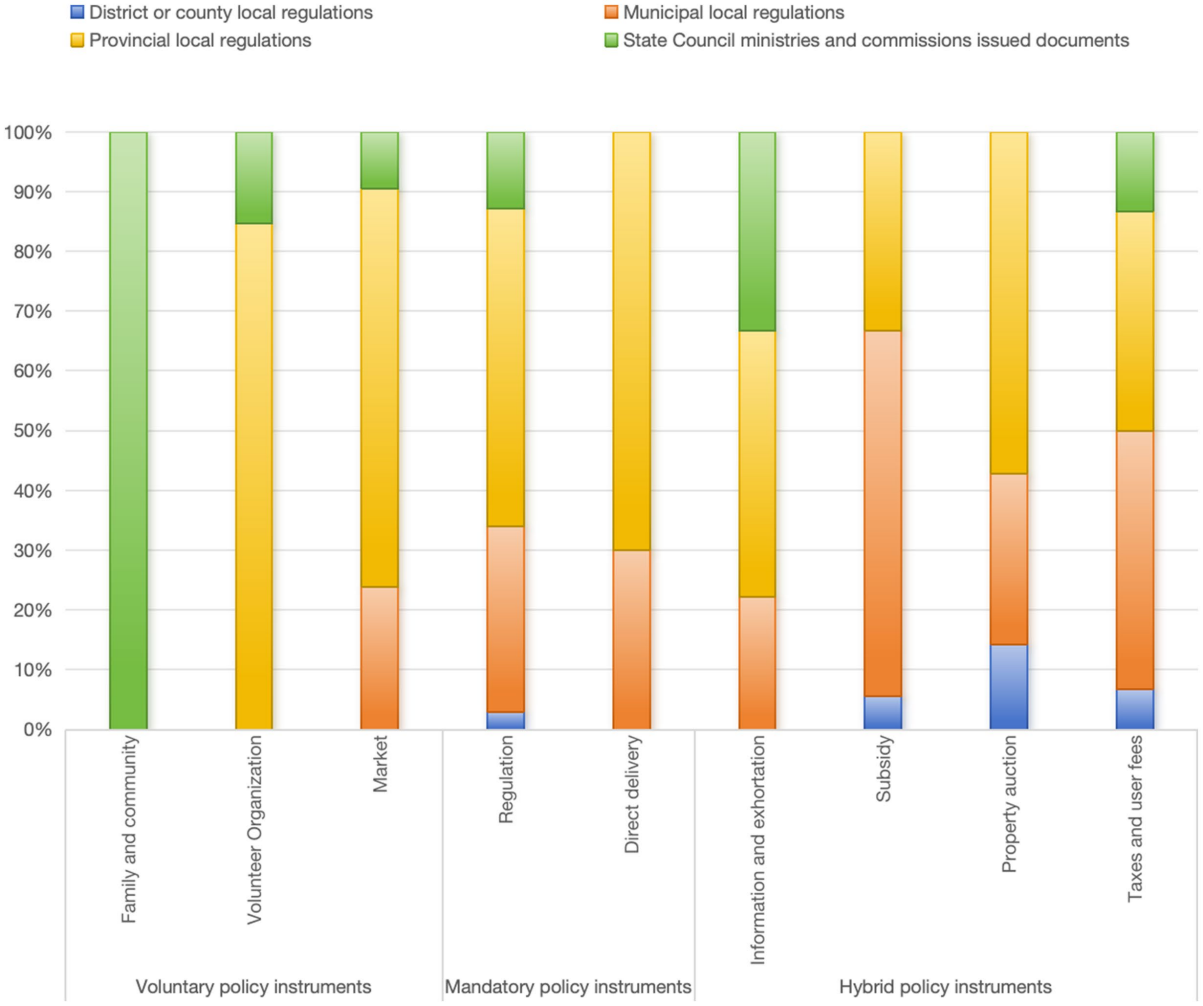
Percentage distribution in X-Y cross analysis.

According to the percentage proportion chart of X-Y cross analysis, as shown in Figure 6, a variety of policy instruments are employed in provincial and municipal local regulations. This indicates that provincial and municipal policy documents have successfully established a connection between the issuance of documents by the State Council and local documents at the district and county levels. This connection underscores the national objective of safeguarding the rights of employees of emerging business sectors, while also enriching and refining the specific content related to occupational injury protection for employees of emerging business sectors. Furthermore, it provides norms and requirements for the implementation of local regulations at the district and county levels. Simultaneously, it highlights the use of control policy instruments to set standards for the documents and reduce uncertainty in their implementation.

### 5.2 X-Z cross analysis

**Figure 7:**
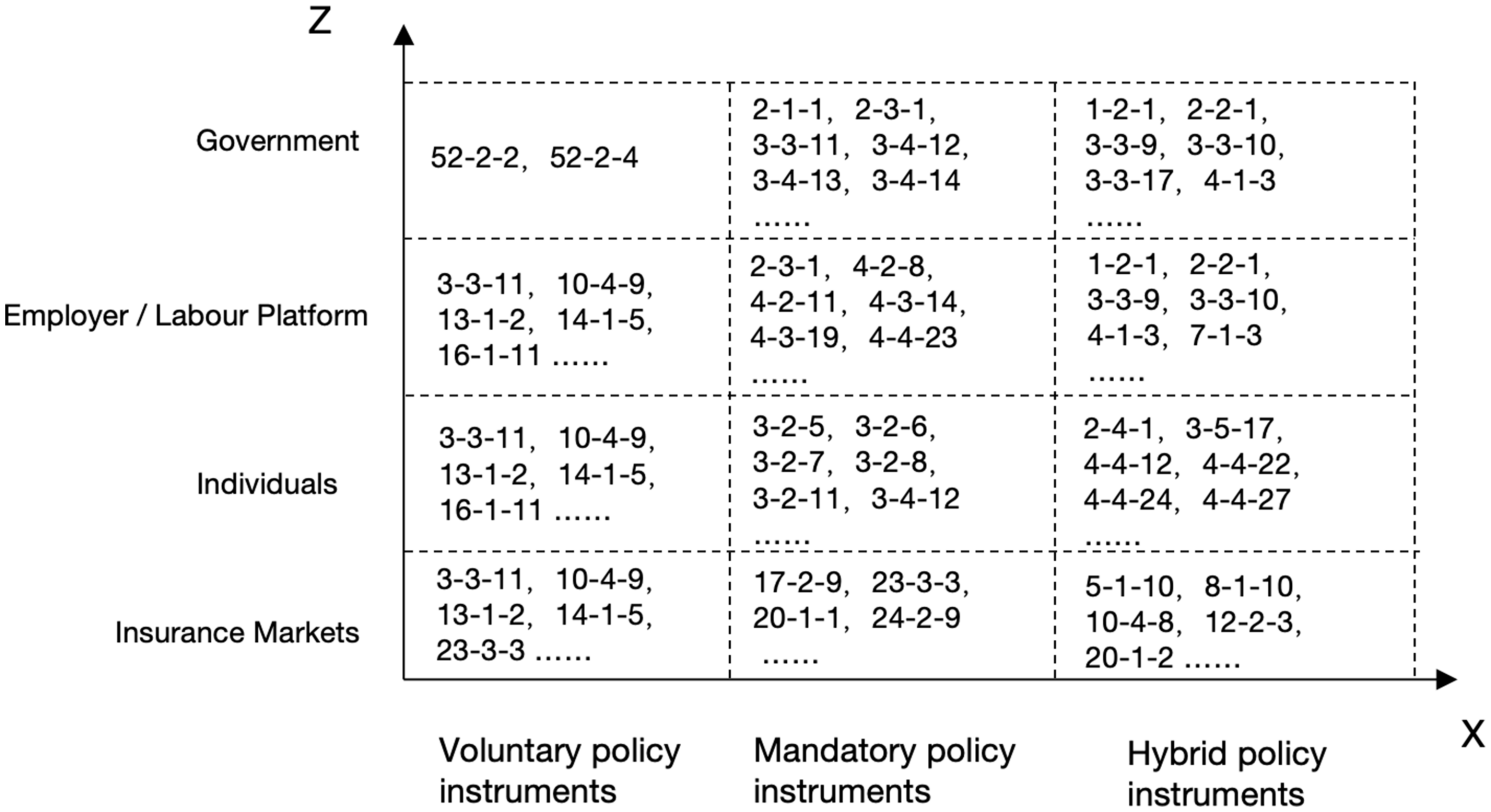
X-Z cross analysis encoding diagram.

The cross X-Z cross analysis can more effectively identify the stakeholders involved with various policy instruments and examine the participation of multiple parties in occupational injury insurance (36). The relevant code is shown in Figure 7, while specific values are presented in Table 7. In the case of mandatory policy instruments, the government, employer/employment platforms, and individuals account for 73.48, 65.2, and 79.25%, respectively. In contrast, the insurance market holds the highest proportion among voluntary policy instruments, accounting for 51.22%. This indicates that mandatory policy instruments primarily engage the government, platforms, and individuals. Through coercive measures, the regulation of these three parties’ behaviors also plays a crucial role in safeguarding their interests. The use of voluntary policy instruments, on the other hand, creates space for the market’s free development, thereby promoting the growth of insurance companies in the area of occupational injury insurance for new types of employees and facilitating the introduction of novel insurance content in alignment with policies to enhance returns.

**Table 7:** Statistics of X-Z cross analysis.

Policy types	Tool names	Government	Employer/Labor platform	Individuals	Insurance markets	Total
Voluntary policy tools	Family and community	2	0	2	0	4
	Volunteer Organization	0	13	13	0	26
	Market	0	21	21	21	63
Mandatory policy tools	Regulation	160	133	224	8	525
	Public enterprise	0	0	0	0	0
	Direct delivery	9	0	9	0	18
Hybrid policy tools	Information and exhortation	8	9	3	5	25
	Subsidies	18	1	18	0	37
	Property auction	4	3	0	7	14
	Taxes and user fees	29	24	4	0	57
Total		230	204	294	41	769
Percentage		100%	100%	100%	100%	100%

**Figure 8:**
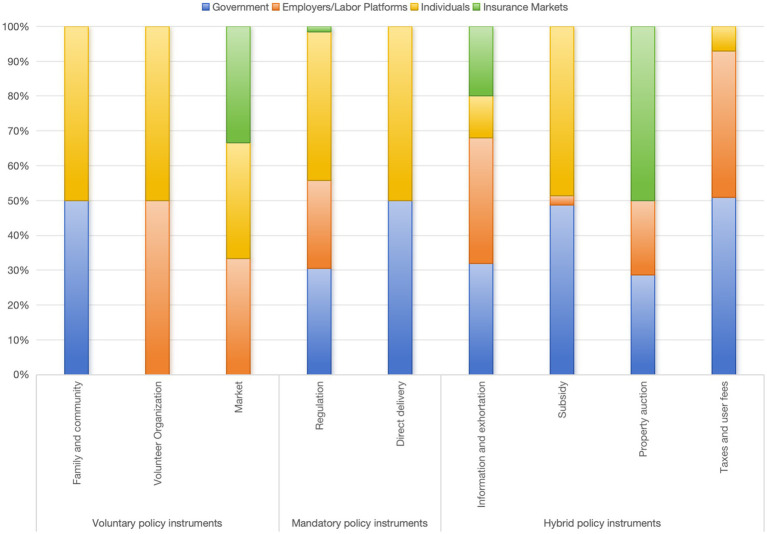
Percentage distribution of X-Z cross analysis.

According to the percentage distribution chart of the X-Z cross analysis (Figure 8), it displays that individuals constitute a significant proportion of stakeholders across all three types of policy instruments. Moreover, this suggests that the implementation of these policy instruments primarily aims to safeguard the legitimate rights and interests of employees of emerging business sectors.

## 6 Conclusion of the policy adaptability

### 6.1 Inequitable utilization of policy instruments and limited adaptability to objectives

In the case of occupational injury insurance, the frequency of use of mandatory policy instruments, especially regulations, is relatively high. This inclination often prioritizes the establishment of norms for the government, platforms, and individuals, while significantly neglecting the involvement of public enterprises. Furthermore, the utilization of hybrid policy instruments remains relatively low, focusing primarily on user payments and taxation without integrating the diverse preferences of multiple stakeholders. Additionally, the insufficient application of voluntary policy instruments results in families and society bearing minimal responsibility, consequently shifting the burden onto the market and fostering an increased dependence on market mechanisms (37).

The imbalanced utilization of policy instruments reflects an imbalance in the construction of policy content, which structure indicates the focal points of a country’s policies (38). On one hand, there is a need to address the deficiencies in occupational injury insurance benefits for employees in emerging business sector. On the other hand, there is an emphasis on fostering linkage and coordination among departments to fulfill their functional responsibilities and provide practical guarantees for employees in these new sectors. However, an excessive reliance on mandatory policy instruments can exacerbate the issues of supply and demand imbalance. Meanwhile, there are differences in policies and their implementation across different regions, making it difficult for employees of emerging business sectors to have a comprehensive understanding of occupational injury insurance participation and claims.

Overall, excessive government intervention leads to an inequitable use of policy instruments and a low degree of flexibility in the overall policy framework. As a result, all stakeholders place too much emphasis on policy objectives and neglect the needs of employees of emerging business sectors when implementing policy directives. This indicates that the country and government have identified the major policy goals during the policy-making and implementation processes, but the use of policy instruments has not achieved a balanced effect.

### 6.2 Although the intensity of policy implementation is exhibiting an increasing trend, the capacity for efficiency requires improvement

The level of policy intensity reflects the country’s emphasis on the emerging business sectors. In the thematic documents issued by various government agencies and departments, multiple perspectives on promoting the economic development of new forms of employment and protecting the labor rights of employees in these sectors have been addressed. Moreover, occupational injury insurance has been stipulated and emphasized, with the policy intensity reaching its peak in 2021. Unfortunately, during the retrieval of policy documents, it was found that only a limited number of policies directly correspond to these themes at both the national and local levels. Furthermore, an examination of the content of policy documents issued by local governments indicates that local transformation has not been achieved, and no detailed rules have been published. Consequently, there may be deviations in the implementation process, raising doubts about their feasibility.

### 6.3 The policy involves multiple stakeholders, but there is a slight deficiency in governance capacity

Numerous stakeholders are involved in the policy, making it challenging to achieve a balance among the interests of various parties (39). Although it has facilitated the participation of multiple entities, including the government, enterprises, platforms, individuals, and the insurance market, the overall effect remains unsatisfactory. In the policy documents related to the protection of employees engaged in emerging business sectors issued by the state, the responsibilities of the government and platforms are clearly delineated. However, there is a lack of detailed procedural steps, necessitating that provincial and local governments supplement their contents. In practice, there are instances where planning is unclear, and documents are copied and utilized without specific regulations tailored to local conditions, resulting in ambiguous implementation. Meanwhile, while the government encourages local administrations to learn from the experiences of pilot cities, numerous challenges arise in promoting and implementing these practices. Therefore, while the policy-making objectives involve multiple stakeholders, the specific capacity for cooperation and governance requires enhancement.

## 7 Suggestions for optimizing policy pathways

As mentioned above, the issue of occupational injury among personnel in emerging business sectors is becoming increasingly prominent. Therefore, it needs to address this issue urgently and achieve the established theoretical goals through the construction of policy optimization pathways. To tackle this issue more comprehensively, this study adopted “content adaptation – instrument rationality – inclusive development” as primary framework. It integrates the three dimensions of the adaptation framework and inclusive development to propose specific policy optimization recommendations, thereby providing more effective solutions for the occupational injury challenges faced by personnel in emerging business sectors.

First, it should enhance the top-level design and expedite the development of a new legal system. To enhance administrative efficiency, the government can implement mandatory measures to regulate actions and expedite the development of a legal system for occupational injury insurance. Drawing from the experiences gained during pilot implementations, it is critical to expand the provisions of China’s Social Security Law concerning occupational injury insurance, particularly focusing on the protection models for new types of personnel. Additionally, it is imperative to clarify the relationship between work injury insurance and occupational injury insurance, and select a protection model that more effectively safeguards the rights and interests of these new employees. Meanwhile, it should strive to establish a new insurance protection sector that deviates from traditional labor models, and explore an occupational injury protection model grounded in socialism with Chinese characteristics. Moreover, it is important to emphasize the mandatory nature of the law, enhance the participation of government entities, platforms, individuals, and insurance companies, and enable them to collaboratively assume the occupational injury risks associated with the new employment models in the evolving industry landscape.

Second, it should standardize policy guidelines, enhance policy refinement, and promote effective implementation. The government should assume an active role in formulating policies, establish relatively unified and clear policy standards, and provide precise explanations for contentious areas in current academic and practical contexts. In particular, it is essential to standardize the definition of insured persons in occupational injury insurance. Presently, there is a tendency for definitions of new employment forms to be unclear and ambiguous. Consequently, the government should provide detailed regulations and explanations when enacting policies. When defining industries and employees, it is also important to develop comprehensive occupational titles and descriptions, continually updating them in accordance with real-world developments. This regulation may empower new employees to independently safeguard their legitimate rights and interests through national government platforms.

Third, it should formulate policy instruments thoughtfully and strive for a balanced application of these instruments (40). Currently, China is in the pilot development stage of occupational injury insurance. In formulating mandatory policy instruments, the government should seek expert advice and assistance from a professional perspective, thereby ensuring a reasonable and balanced distribution of these instruments. Specifically, given the excessive reliance on mandatory policy instruments and the limited involvement of public enterprises in this process, the government can collaborate with state-owned enterprises and implement policy-based pilot demonstrations to provide experiential references for developing occupational injury insurance. Regarding hybrid policy instruments, it needs to modify, adjust, and update them appropriately in collaboration with stakeholders, ensuring they are well-planned and aligned with policy objectives. For voluntary policy instruments, it is crucial to strengthen the roles of families and society. Meanwhile, community members can make their contributions by assisting employees of emerging business sectors in participating in occupational injury insurance.

Fourth, it should regularly assess the effectiveness of the pilot program and strive to gradually enhance its implementation, thereby continuously promoting the development of occupational injury insurance in China. Regular assessments of pilot cities can be conducted through methods such as summarizing pilot status reports, distributing questionnaires regarding occupational injury insurance participation among employees of emerging business sectors in pilot cities, and analyzing data to ensure efficacy. If the pilot outcomes are favorable and can be implemented nationwide, the scope of policy implementation will be gradually expanded to include a greater number of employees of emerging business sectors. Conversely, if the pilot outcomes are unsatisfactory, we should identify the underlying issues and implementing corrective measures, thereby drawing the attention of other pilot cities and formulating specific regulations to prevent the recurrence of such problems. The ultimate goal of the pilot program is to explore a more suitable occupational injury protection model for employees in the emerging business sectors.

Fifth, it should take into account the demands of employment and continuously develop strategies in response to evolving needs (41). When formulating comprehensive occupational injury insurance policies, the government should consider public sentiment regarding current pressing issues, conduct preliminary investigations into the needs of employees in emerging business sectors, and ensure that policies are adequately supplied based on these needs. Specifically, regarding the scope of application and recognition of occupational injury insurance for employees of emerging business sectors, local governments should develop regulations informed by existing laws and regulations that protect the rights of employees, while also addressing the needs of employees in these new sectors. Meanwhile, the government should prioritize the benefits of insured individuals, as well as supervise medical and health institutions to ensure the provision of essential services and facilitate enterprise platforms in fulfilling their designated functions, thereby enhancing the scope of protection. Additionally, the government can implement a scoring system and deploy targeted questionnaires in grassroots services to regulate the conduct of service personnel and assess the needs of employees in emerging business sectors. Accordingly, it may enhance the quality and effectiveness of official services, improve the satisfaction of employees of emerging business sectors with their insurance, and encourage active participation in the insurance system. Furthermore, the government should function as the security fund pool, increase both the amount and intensity of protection, and establish supplementary insurance rates to ensure that funds are allocated and used legitimately.

Finally, it should strengthen the promotion of policies and enhance the capacity to address occupational injury risks. Policy promotion is a crucial mechanism for the implementation of policies (42). In this regard, it needs to conduct research to assess the public’s satisfaction with these policies. Currently, the understanding of occupational injury insurance policies among employees in new employment formats is insufficient. Therefore, the government should engage society, enterprises, and other stakeholders in promoting occupational injury insurance for new forms of employment, while increasing personnel’s awareness of insurance participation. Through grassroots efforts and offline community outreach, combined with online media and other digital instruments, government can develop clear and accessible policy interpretation articles or videos to effectively convey information to such population, thereby attracting their attention. For industries that pose significant risks to physical health, it should organize centralized training sessions. If necessary, tailored safety regulations specific to certain industries can be issued to maximize the protection of the rights of employees of emerging business sectors and safeguard their interests. Moreover, various industries should be encouraged to organize targeted lectures, develop specific guidelines, teach prevention methods, improve risk response capabilities, prioritize the safety of employees of emerging business sectors, and acknowledge the importance of social insurance.

## Data Availability

The raw data supporting the conclusions of this article will be made available by the authors, without undue reservation.
